# Effect of Molybdenum on Microstructural Evolution and High Cycle Fatigue Properties of Ti-xMo-2Fe Alloys

**DOI:** 10.3390/ma19010010

**Published:** 2025-12-19

**Authors:** HyoWoon Hwang, Dong-Geun Lee

**Affiliations:** 1 Ceramic Materials R&D Center, OSSTEM IMPLANT Co., Ltd., Seoul 07789, Republic of Korea; hyowoon7501@naver.com; 2 Department of Materials Science and Metallurgical Engineering, Sunchon National University, Suncheon 57922, Republic of Korea

**Keywords:** Ti-Mo-Fe alloy, high cycle fatigue, α″ martensite, ω phase

## Abstract

Ti-xMo-2Fe alloys with high specific strength were designed by adding Mo and Fe as β-stabilizing elements. The influence of cold swaging on the martensitic transformations in Ti-xMo-2Fe (x = 3.4, 5, 9.2 wt.%) alloys was investigated. In these alloys, appropriate chemical compositions promote a stress-induced phase transformation from the β phase to orthorhombic α″ martensite, which improves elongation while maintaining high strength. As the Mo content increases from 3.4 to 5 wt.%, the amount of β-stabilizing elements increases and the β stability is enhanced, thereby altering the phase transformation mechanism. In the Ti-9.2Mo-2Fe alloy, both α″ martensite and a very hard ω phase were identified by X-ray diffraction and transmission electron microscopy. The hard and brittle ω phase causes premature brittle fracture prior to macroscopic yielding. Among the investigated alloys, the Ti-5Mo-2Fe alloy exhibits the best overall combination of high tensile strength, elongation to failure, and high fatigue strength.

## 1. Introduction

Titanium alloys possess high specific strength, excellent corrosion resistance, and good biocompatibility and are therefore widely used as essential structural materials in various fields such as aerospace, marine engineering, and biomedical applications [1,2]. Ti undergoes allotropic transformation with a hexagonal close packed (HCP) α phase at room temperature and a body centered cubic (BCC) β phase at 882 °C and above [3,4]. A previous study showed that the crystal structures of titanium alloys are stabilized by the addition of certain alloying elements [5]. In the case of β-Ti alloys, β stabilization elements are added so that their β phases, which have stable BCC structures at high temperatures and are stably maintained at room temperature, and these alloys exhibit isotropic mechanical behaviors and excellent workability due to their BCC crystal structures [6].

Cotton et al. [7] used molybdenum equivalency (MoE) to classify β-Ti alloys into three types, and according to Mo [8], MoE is a major parameter for comprehensively evaluating how β phase stabilization is affected by α phase and β phase stabilizing elements as well as neutral elements. Near β-Ti alloys have an MoE that is greater than or equal to 5.0 but less than or equal to 10.0, whereas metastable β-Ti alloys have an MoE that is ≥10.0 and ≤30.0, and stable β-Ti alloys have an MoE that is >30.0 [9]. Here, β-stabilizing elements are divided into two types: isomorphic and eutectoid [10]. Isomorphic elements include Mo, V, Ta, Zr, Hf, Nb, and Re, and eutectoid elements include Cr, Mn, Fe, Co, Ni, and Cu. In the case of isomorphic elements, the alloys form complete solid solution, and in the case of eutectoid elements, two or more phases exist [11]. However, most β-Ti alloys have poor price competitiveness as compared with other commercial alloys because they include expensive alloying elements such as Ta, Nb, and Zr [12]. Therefore, intensive research is being conducted on reducing the use of expensive alloying elements and replacing them with inexpensive alloying elements such as Fe, Mn, and Cr. Accordingly, alloys such as Ti-Mn-Fe [13], Ti-Cr-Sn-Zr [13], and Ti-Fe-Ta-Zr [14] have been developed.

This study designed a metastable β-Ti alloy by adding the relatively inexpensive alloying elements Fe and Mo instead of expensive alloying elements such as Ta, Nb, or Zr. Previous alloy-design studies have suggested that combining an isomorphic β stabilizer such as Mo with a eutectoid β stabilizer such as Fe is an efficient way to tune β stability and deformation mechanisms while reducing the overall amount of expensive alloying elements [15,16,17]. Fe is a strong β-stabilizing element that can effectively strengthen Ti alloys when added. Fe exhibits a fast diffusion rate in titanium, which promotes homogenization, and provides a pronounced solid solution strengthening effect that further enhances the mechanical strength of the alloy. In addition, Fe is an essential element in the human body. Fe-containing β-Ti alloys have been reported to exhibit acceptable cytocompatibility when the Fe content and ion release are properly controlled; however, excessive Fe ion release may cause cytotoxic effects, and careful control of alloy composition and microstructure is therefore required [18,19]. Mo is a non-toxic and non-allergenic element that enhances the corrosion resistance of titanium alloys, and this effect is further strengthened by the formation of MoO_3_, which provides an additional protective layer. Moreover, Mo exhibits a slow diffusion rate at high temperatures, contributing to improved high-temperature strength and mechanical stability of the alloy [20,21]. On this basis, the Ti-xMo-2Fe (x = 3.4, 5, 9.2 wt.%) system was selected in the present work as a model alloy family that exploits the cost-effectiveness of Fe and the biocompatible, corrosion-resistant character of Mo.

In the case of metastable β-Ti alloys, previous studies have shown that the strength and toughness of the designed alloy can be optimized mainly by controlling the alloy microstructure, and the importance of metastable β-Ti alloys has steadily grown [22,23]. It has been reported that the deformation of β-Ti alloys occurs through various mechanisms such as dislocation slip, twinning, and stress-induced martensitic (SIM) transformation based on β stability [24]. The lower the β stability, the greater the extent of the SIM transformation. As β stability increases, more dislocation slip and twinning occur [25]. Therefore, a detailed understanding of the relationship between β stability and SIM transformation is necessary.

This study designed a metastable β-Ti alloy by adding relatively inexpensive Fe and Mo to ensure price competitiveness. The study also used SIM formations to develop a metastable β-Ti alloy that has high strength and high formability. Finally, this study observed the manner in which Mo content affects the microstructures and mechanical properties of Ti-Mo-Fe alloys.

## 2. Experimental Procedures

### 2.1. Alloy Design

The d-electron alloy design method [26,27] is based on cluster DV-Xα and uses bond order (*Bo*) and metal d-orbital energy level (*Md*) values to provide information on the phase stability and transformation mechanisms of titanium and alloying elements. *Bo* refers to the covalent bond strength between the titanium and alloying element, and *Md* refers to the average orbital energy in terms of electronegativity and the element radius. These two parameters can be calculated using Equation (1).

(1) $$\bar{Bo}=\sum_{i=1}^{n}X_{i}\cdot {\left(Bo\right)}_{i},\;\bar{Md}=\sum_{i=1}^{n}X_{i}\cdot {\left(Md\right)}_{i}$$

Here, Xi is the atomic fraction of the alloying element that is the ith component, and ${\left(Bo\right)}_{i}$ and ${\left(Md\right)}_{i}$ are the respective values for the ith component. The Mo contents of 3.4, 5, and 9.2 wt.% were selected so that the alloys represent different levels of β stability in terms of the Bo–Md, MoE, and e/a design parameters. The Ti-3.4Mo-2Fe alloy is located in a region where an α + β microstructure is stabilized, whereas the Ti-5Mo-2Fe alloy lies in an intermediate β-stability region where stress-induced α″ martensite can form. The Ti-9.2Mo-2Fe alloy is positioned in a high β-stability region in which the formation of the ω phase has been reported. Therefore, these three compositions cover a stepwise change in β stability, allowing a systematic comparison of the differences in microstructure and mechanical properties as β stability varies.

Basic design information is provided in Table 1. The phase stability and deformation of the designed titanium alloys could be predicted using the electron per atom (e/a) ratio. A previous study showed that β stability increases as the e/a ratio increases, and twinning occurs at e/a ratios between 4.10 and 4.20, whereas dislocation slip occurs at e/a ratios between 4.20 and 4.27 [28]. Another study showed that the ω phase occurs within the β matrix at e/a ratios between 4.13 and 4.30 [29]. In addition, MoE is a value that shows the effects of alloying elements on β-phase stabilization, and a previous study reported that when the MoE value is greater than 8, the β phase becomes metastable and an SIM transformation of β → $\alpha^{\prime \prime }$ occurs under applied mechanical deformation stress [22]. However, because MoE is limited solely to SIM transformation predictions, predicting the occurrence of other transformation mechanisms is difficult.

This study fabricated Φ 16 ingots of a Ti-xMo-2Fe (x = 3.4, 5, 9.2) alloy with Ti (cp-Ti, 99.9%), Mo (99.95%), and Fe (99.5%) as raw materials using a non-consumable vacuum arc re-melting technique. The fabricated ingots underwent a solution heat treatment at 850 °C for 1 h and then were furnace-cooled to room temperature. Next, the solution heat-treated Φ 16 ingots were transformed into Φ 11 swaged specimens through cold swaging. It resulted in an area reduction of approximately 52.7% (true strain ≈ 0.75).

The Ti-xMo-2Fe (x = 3.4, 5, 9.2 wt.%) alloys may be effective at reducing the cost of raw materials as compared with other commercial and β-Ti alloys. Compared to the widely used Ti-6Al-4V, Ti-39Nb-6Zr (TNZ40), and Ti-29Nb-13Ta-4.6Zr alloys, reductions of 78.2–78.8%, 59.0–60.6%, and 59.6–60.6% were calculated, respectively [30], and these results showed that replacing expensive alloying elements with inexpensive Mo and Fe was effective at reducing material costs.

### 2.2. Microstructural Analysis

The solution heat-treated and cold-swaged alloys were designated as Ti-3.4Mo-2Fe (32 SW or below), Ti-5Mo-2Fe (52 SW or below), and Ti-9.2Mo-2Fe (92 SW or below). To compare and analyze the microstructures of the designed alloys, the specimens were cut perpendicular to their lengths and hot mounted, and micro-polishing was performed using #220–#2000 sandpaper, 3 μm and 1 μm abrasives, and colloid silica. Next, etching was performed by mixing 2 mL of nitric acid (${\mathrm{HNO}}_{3}$) and 2 mL of hydrofluoric acid (HF) in 100 mL of distilled water ($H_{2}O$). To observe changes in the alloy microstructures based on the Mo content, this study employed optical microscopy (OM, BX53M, Olympus, Tokyo, Japan), scanning electron microscopy (SEM, JSM-7100F, JEOL, Tokyo, Japan), and electron backscattered microscopy (JSM-7100F, JEOL). In addition, transmission electron microscopy (TEM, JEOL 2100F, JEOL) operating at 200 kV was used to observe the $\alpha^{\prime \prime }$ phase formed by SIM transformation that occurs during cold swaging. X-ray diffraction (XRD) analysis was performed using a D8 Discover diffractometer (Karlsruhe, Germany) operating at 40 kV and 30 mA under Cu Kα radiation, and Equation (2) that follows was used to calculate the $\alpha ,\alpha^{\prime \prime },\;\beta \;and\;\omega \;$ phase fractions. No correction for preferred orientation/texture was applied in the XRD-based phase-fraction analysis; therefore, the reported phase fractions should be regarded as semi-quantitative.

(2) $$V_{f,\alpha }=\frac{A_{\alpha }}{A_{\alpha }+A_{a^{\prime \prime }}+A_{\beta }+A_{\omega }},\;V_{f,a^{\prime \prime }}=\frac{A_{a^{\prime \prime }}}{A_{\alpha }+A_{a^{\prime \prime }}+A_{\beta }+A_{\omega }},\;V_{f,\beta }=\frac{A_{\beta }}{A_{\alpha }+A_{a^{\prime \prime }}+A_{\beta }+A_{\omega }}\;V_{f,\omega }=\frac{A_{\omega }}{A_{\alpha }+A_{a^{\prime \prime }}+A_{\beta }+A_{\omega }}$$

where $V_{f,\alpha }$, $V_{f,a^{\prime \prime }}$, $V_{f,\beta }$ and $V_{f,\omega }$ are the volume fractions, and $A_{\alpha }$, $A_{a^{\prime \prime }}$, $A_{\beta }$ and $A_{\omega }$ are the total areas for the α, $\alpha^{\prime \prime },\;\beta$ and ω phases, respectively.

### 2.3. Mechanical Evaluation

To evaluate the alloy mechanical properties at room temperature, this study performed Vickers hardness, room temperature tensile, and high-cycle fatigue tests. For the hardness tests, a Vickers hardness tester (HM-200, Mitutoyo, Tokyo, Japan) was used to measure 12 points under a 1-kgf load from the circumference of the alloy specimens toward the center, and average values were calculated, including the standard deviation and excluding the minimum and maximum values.

**Figure 1:**
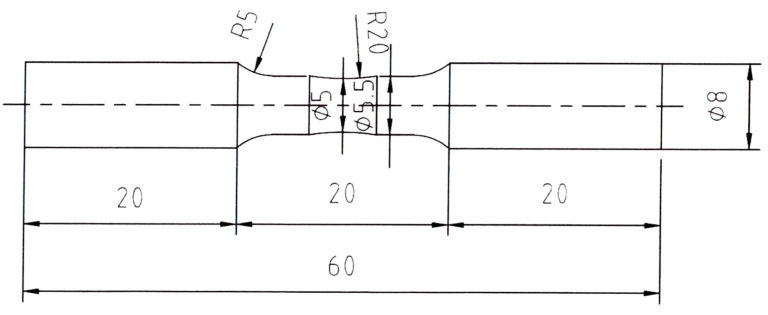
Schematic diagram of the fatigue test specimen.

For the tensile tests, tensile specimens were fabricated through electric discharge machining at the ASTM E8/E8M subsize standard, where the specimens had a total length of 100 mm, gauge length of 25 mm, thickness of 6 mm, and width of 3 mm. The tests were performed on three specimens at a strain rate of 1 × 10^−3^/s at room temperature using a universal testing machine (BESTUTM-10MD, SSaul Bestech, Seoul, Republic of Korea). To evaluate the high-cycle fatigue properties of the designed alloys, fatigue specimens with Φ 5 centers were fabricated, and their surfaces were polished with 3- and 1-µm abrasives. A rotation bending fatigue tester (KDMT-240, KDMT, Siheung, Republic of Korea) was used to perform 10^7^ rotations under various stress conditions using four specimens (Figure 1), with the tests conducted under rotating bending loading at stress levels ranging from 300 to 550 MPa and a stress ratio of R = −1.

## 3. Results and Discussion

### 3.1. Microstructural Characterization

**Figure 2:**
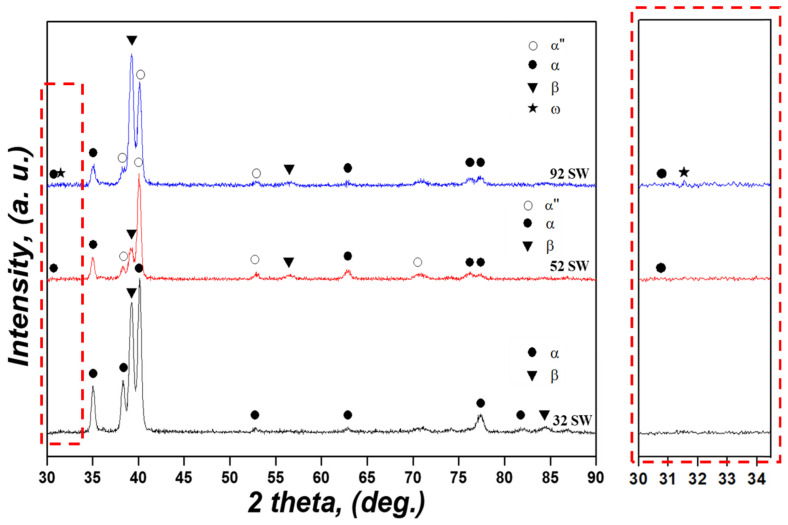
X-ray diffraction patterns of the as-swaged Ti-xMo-2Fe alloys.

XRD patterns of the Ti-xMo-2Fe (x = 3.4, 5, 9.2 wt.%) alloys are shown in Figure 2. Fe is known to be a eutectoid element that forms TiFe intermetallic compounds [31]. However, TiFe peaks were not observed in any of the alloys due to their low Fe content [15]. In addition, α-phase peaks were observed in all specimens due to the slow cooling rate, and an α″ phase peak was observed in 52 SW, whereas α″ peaks and a very weak diffraction feature suggestive of the presence of an ω phase were observed in 92 SW. This resulted from SIM transformation from the β phase to the α″ phase derived from the stress that occurred during cold swaging. However, this did not occur in 32 SW, which has low β stability, because the α″ and ω phases depend on β stability [32]. In addition, 92 SW was uniquely within the e/a range (4.13–4.30) in which the ω phase was formed, and a previous study showed that the ω phase is formed at a high pressure [33]. In short, a transformation from the β to the ω phase occurred in 92 SW during cold swaging. By combining the MoE and e/a values listed in Table 1 with the phase fractions obtained from XRD (Table 2), the correlation between changes in β stability with alloy composition and the corresponding transformation behavior can be clarified more clearly. The 32 SW and 52 SW alloys lie in a relatively low MoE and e/a range, such that an α + β microstructure is stabilized and only a small amount of α″ martensite is formed during cold swaging, whereas the 92 SW alloy is located in a high β-stability region beyond the critical MoE and e/a values, corresponding to a compositional range in which the ω phase can form during cold swaging. These results indicate that as β stability increases, the transformation path changes from α + β to α″ and finally to α″ with possible ω formation, as suggested by the weak diffraction feature and the small estimated ω fraction (~2 vol%).

Phase volume fractions measured through the XRD patterns lists in Table 2. As previously mentioned, 32 SW has relatively low β stability and thus exhibited the highest α phase fraction ($V_{f,\alpha }=66.4\%$). In addition, the $\alpha^{\prime \prime }$ phase did not form in 32 SW because SIM transformation did not occur during cold swaging. As the Mo content increased, the α phase fraction ($V_{f,\alpha }=23.9\%)$ decreased in 52 SW, and the $\alpha^{\prime \prime }$ phase ($V_{f,\alpha^{\prime \prime }}=57.2\%)$ was formed due to SIM transformation. 92 SW, which had the highest added Mo content, also had the lowest α phase fraction ($V_{f,\alpha }=12.6\%)$ due to its high β stability, and the ω phase ($V_{f,\omega }=2\%)$ was formed. However, 32 SW had a higher β phase fraction ($V_{f,\beta }=33.6\%)$ than that of 52 SW ($V_{f,\beta }=18\%)$ despite having the lowest β stability. SIM transformation occurred above a certain range of β stability, and 32 SW showed the highest β phase fraction. This was because SIM transformation did not occur due to the low β stability of 32 SW, whereby the β phase did not transform to the $\alpha^{\prime \prime }$ phase. This meant that β stability varied according to Mo content, and SIM transformation occurred above a certain range.

**Figure 3:**
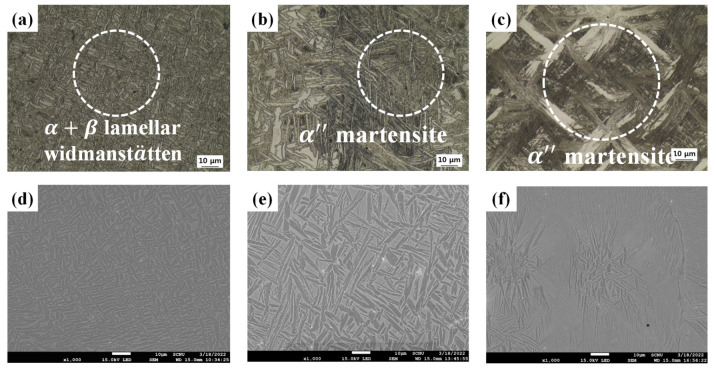
OM micrographs of the as-swaged Ti-xMo-2Fe; (**a**) 32 SW, (**b**) 52 SW and (**c**) 92 SW and SEM micrographs of the Ti-xMo-2Fe; (**d**) 32 SW, (**e**) 52 SW, and (**f**) 92 SW.

**Figure 4:**
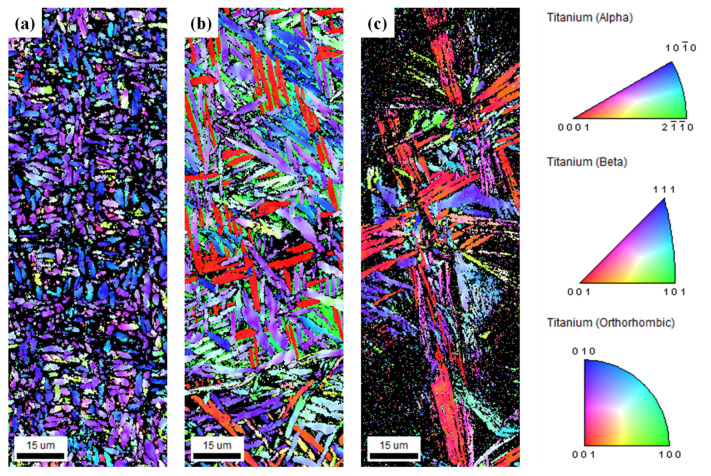
Inverse pole figure (IPF) maps of the as-swaged Ti-xMo-2Fe; (**a**) 32 SW, (**b**) 52 SW, and (**c**) 92 SW.

Figure 3 shows the microstructures of the Ti-xMo-2Fe (x = 3.4, 5, 9.2 wt.%) alloys observed by OM and SEM. In the actual 32 SW, the Widmanstätten structure was observed due to the slow cooling rate and consisted of an α lamellar (dark phase) with a width of 1.35 µm and a β phase (white phase). The $\alpha^{\prime \prime }$ phase did not form through SIM transformation during cold swaging because of low β stability. In 52 SW and 92 SW, the α phase occurred due to the slow cooling rate, and SIM transformation occurred due to relatively high β stability such that lath-shaped $\alpha^{\prime \prime }$ phases with respective widths of 0.93 and 0.48 µm were observed. It is reported that the width of the α-lath affects high-cycle fatigue (HCF) strength and is a critical factor [34]. Figure 4 shows an inverse pole figure map for analyzing differences in crystal orientation based on Mo content. It was observed that 32 SW mostly had a ${<10\bar{1}0>}_{\alpha }$ texture, whereas 52 SW and 92 SW had ${<001>}_{\alpha^{\prime \prime }}$ textures. This was due to the fact that, as reported in previous studies, the $\alpha^{\prime \prime }$ phase is correlated with the ${\left\{110\right\}}_{\beta }//{\left\{001\right\}}_{\alpha^{\prime \prime }}$ and ${<111>}_{\beta }//{<101>}_{\alpha^{\prime \prime }}$ crystal orientations [35], and β stability increases as Mo content increases such that the $\alpha^{\prime \prime }$ phase is formed as a result of SIM transformation during cold swaging.

**Figure 5:**
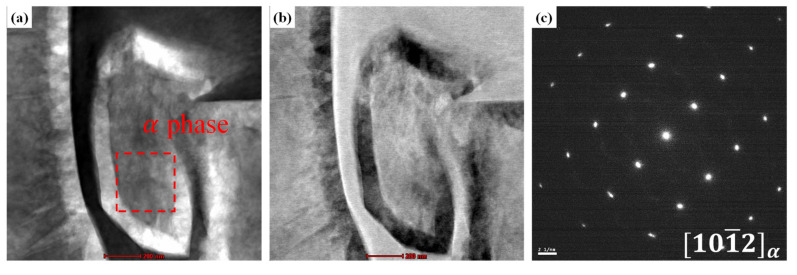
TEM micrographs of 32 SW; (**a**) BF-TEM image, (**b**) HAADF image, and (**c**) DF-TEM image.

**Figure 6:**
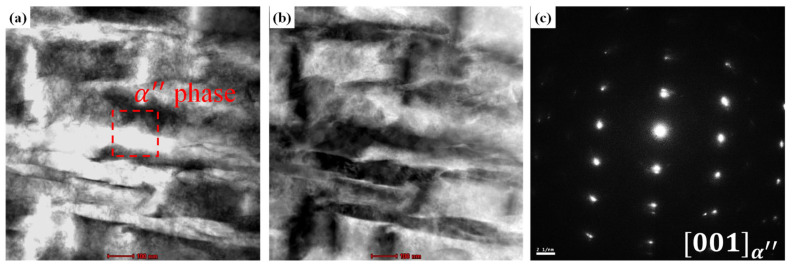
TEM micrographs of 52 SW; (**a**) BF-TEM image, (**b**) HAADF image, and (**c**) DF-TEM image.

**Figure 7:**
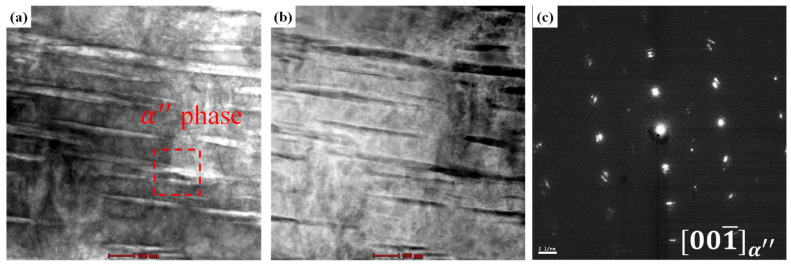
TEM micrographs of 92 SW: (**a**) BF-TEM image, (**b**) HAADF image, and (**c**) DF-TEM image.

To directly examine the $\alpha^{\prime \prime }$ phase that formed as a result of SIM transformation, the 32 SW, 52 SW, and 92 SW alloys were observed under TEM, and the results are shown in Figure 5, Figure 6 and Figure 7. Figure 5a,b are bright-field and a high-angle annular dark-field images of 32 SW, and Figure 5c shows the selected-area diffraction pattern of the corresponding region. In 32 SW, the α phase pattern was observed at z = $\left[10\bar{1}2\right]$. Simultaneously, we confirmed that 32 SW had the α phase due to its slow cooling rate and lowest β stability. Figure 6a–c are TEM images of 52 SW. In 52 SW, the $\alpha^{\prime \prime }$ phase pattern was observed in the 20 nm lath in the region shown in Figure 6a. Figure 7a–c are TEM images of 92 SW. In the region shown in Figure 7a, the $\alpha^{\prime \prime }$ phase pattern was found in the 6 nm lath, which was narrower than that in 52 SW.

**Figure 8:**
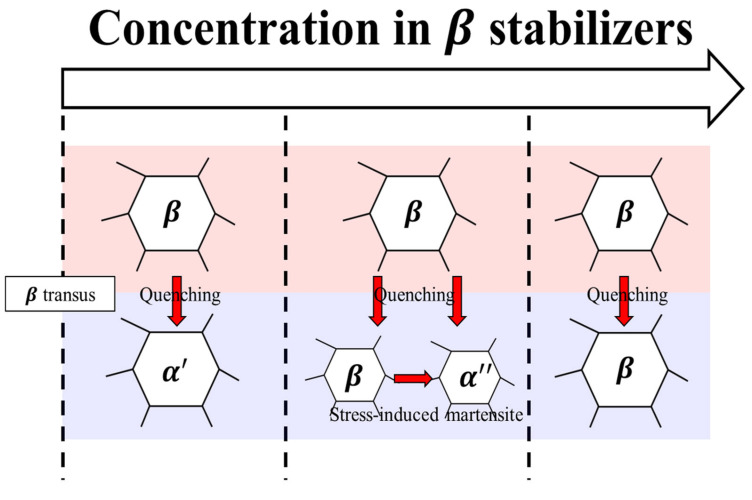
The change in microstructure in concentration of beta stabilizing elements in the titanium alloy.

Microstructural observations did not reveal the $\alpha^{\prime \prime }$ phase in 32 SW, but it was observed in 52 SW and higher. Because the $\alpha^{\prime \prime }$ phase was observed when Mo content was 5 wt.% or greater, a critical concentration of β stability was present to form the $\alpha^{\prime \prime }$ phase through SIM transformation. Therefore, the $\alpha^{\prime \prime }$ phase did not form in 32 SW due to its slow cooling rate and insufficient β stability for phase transformation. Conversely, in the case of 52 SW and 92 SW, the $\alpha^{\prime \prime }$ phase formed because of SIM transformation during cold swaging due to β stability when the Mo content was above a certain level (Figure 8).

### 3.2. Mechanical Properties

**Figure 9:**
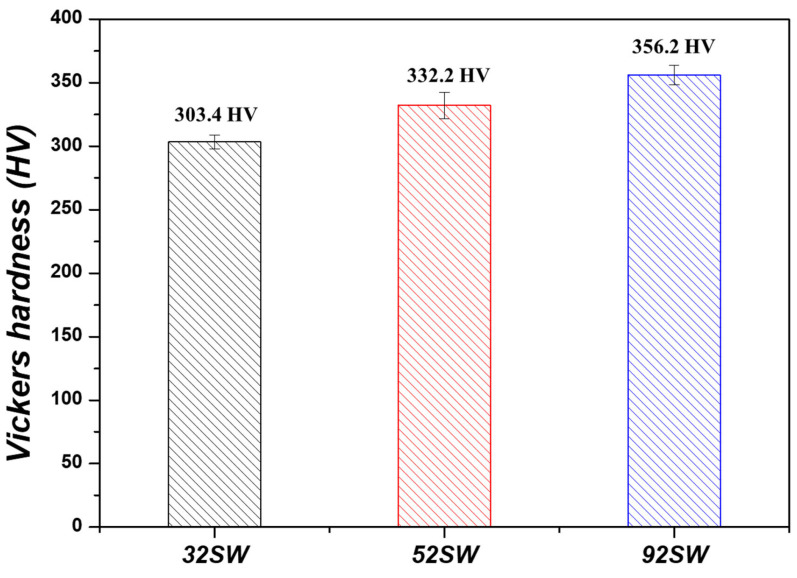
Vickers hardness of the as-swaged Ti-xMo-2Fe alloys.

Figure 9 shows the changes in the Vickers hardness of the Ti-xMo-2Fe (x = 3.4, 5, 9.2 wt.%) alloys, where 32 SW, 52 SW, and 92 SW had hardness values of 303.4, 333.2, and 356.2 HV, respectively, confirming that the hardness values increased as the Mo content increased. A previous study showed that hardness values change in the order of $H_{\omega }>H_{\alpha^{\prime }}>H_{\alpha^{\prime \prime }}>H_{\beta }>H_{\alpha }$ [36]. In our study, 32 SW had the lowest hardness value because it consisted solely of the Widmanstätten structure with an α+β lamellar form, which is a relatively soft structure. 52 SW, which experienced an increase in Mo content, also experienced an increase in hardness due to the fact that the $\alpha^{\prime \prime }$ phase formed as a result of SIM transformation, along with the Widmanstätten structure that had an α+β lamellar form. However, 92 SW, which had the highest Mo content, had a higher hardness value even though its $\alpha^{\prime \prime }$ phase fraction was reduced as compared with 52 SW. 92 SW had the highest hardness due to the fact that a very hard ω phase formed as β stability increased.

**Figure 10:**
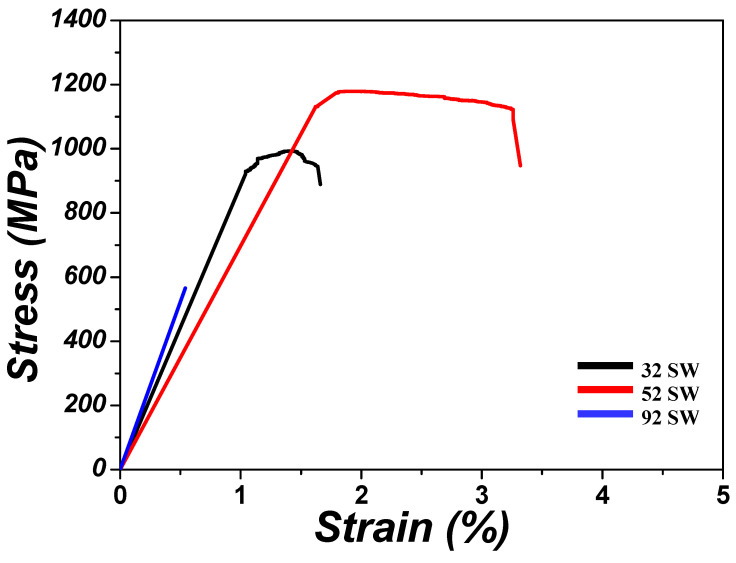
Tensile stress–strain curves of the as-swaged Ti-xMo-2Fe alloys.

The stress–strain curves obtained by the room-temperature tensile tests performed on the Ti-xMo-2Fe (x = 3.4, 5, 9.2 wt.%) alloys are shown in Figure 10. Table 3 lists the specific tensile property values measured from these curves for yield strength, ultimate tensile strength, and elongation. 32 SW exhibited a yield strength of 969.0 MPa, ultimate tensile strength of 993.2 MPa, and elongation of 1.7%. 52 SW had the highest yield and ultimate tensile strengths of 1146.4 MPa and 1179.4 MPa, respectively, and also exhibited the highest elongation at 3.3%. 92 SW, which had the highest Mo content, fractured prior to the yield point, and it had the lowest tensile strength and elongation at 565.8 MPa and 1.1%, respectively. The mechanical properties of titanium alloys are closely related to the phases and phase fractions that make up the alloys [37], and understanding the constituent phases of each alloy is necessary. 32 SW consisted of the α phase ($V_{f,\alpha }=66.4\%)$ and β phase ($V_{f,\beta }=33.6\%)$ phases, but 52 SW consisted of the α phase ($V_{f,\alpha }=23.9\%)$, β phase ($V_{f,\beta }=18.9\%)$, and $\alpha^{\prime \prime }$ ($V_{f,\alpha^{\prime \prime }}=57.2\%)$ phases due to SIM transformation. As the Mo content increased, the formation of the α phase, which has few slip systems, was suppressed, and elongation increased. In addition, when Mo was added, tensile strength increased due to the solid solution strengthening effect. Therefore, 52 SW exhibited a higher elongation and tensile strength than 32 SW. However, in the case of 92 SW, brittle fracture occurred prior to the yield point even though it had the highest amount of added Mo. This fracture occurred because the ω phase formed within the β matrix due to the high β stability, and the ω phase led to brittle fracture [38].

**Figure 11:**
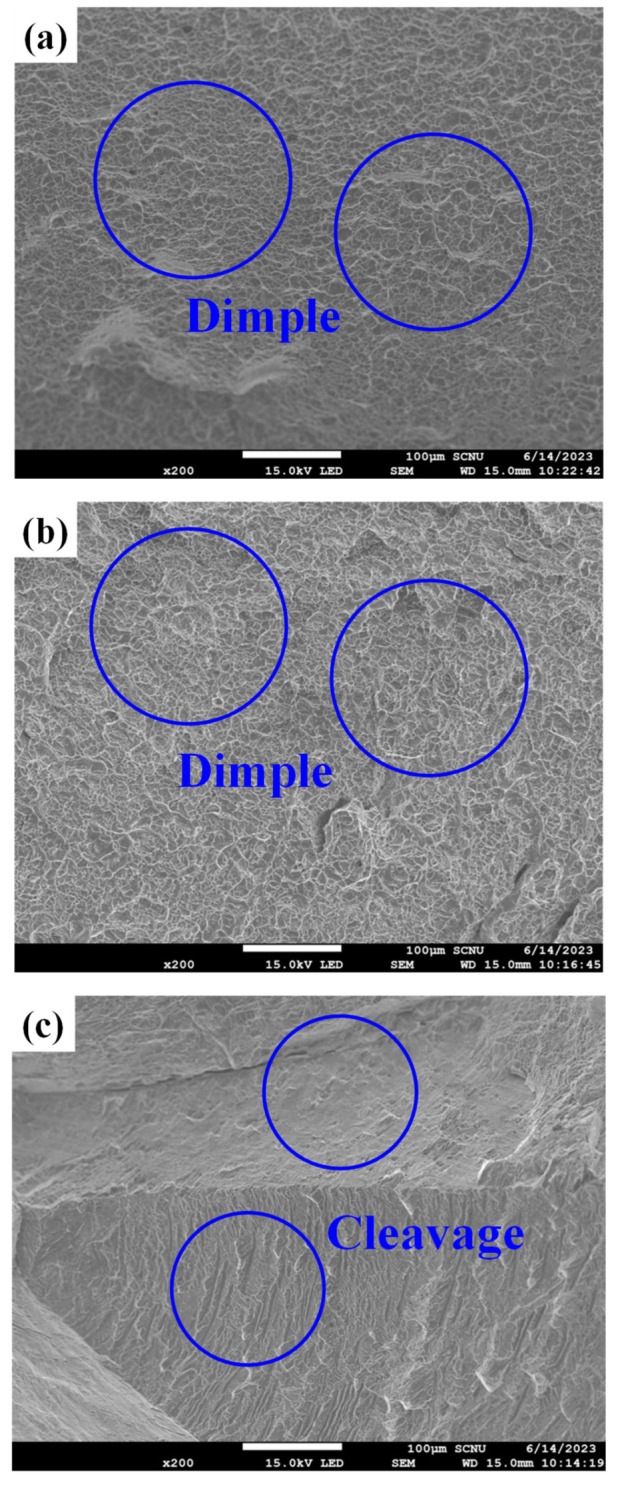
SEM fractographs of the as-swaged Ti-xMo-2Fe alloys after tensile test: (**a**) 32 SW, (**b**) 52 SW and (**c**) 92 SW.

Figure 11 shows the fracture surfaces of the fractured tensile specimens. As shown in Figure 11a,b, dimple patterns as a characteristic of ductile fracture behavior have been observed on the fracture surface of 32 SW and 52 SW. However, in case of the 92 SW (Figure 11c), the cleavage fracture surface was observed, which is a typical characteristic of brittle fracture behavior.

**Figure 12:**
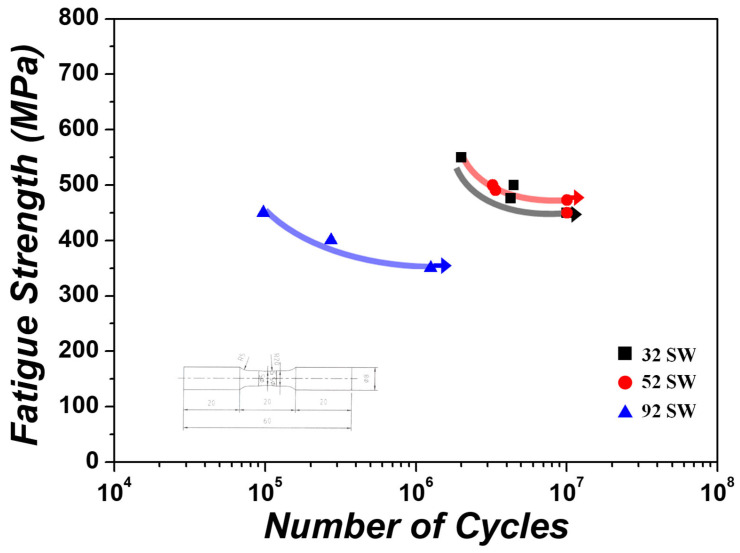
High-cycle fatigue S-N curves of the as-swaged Ti-xMo-2Fe alloys.

The stress—the number of rotation curves (S-N curves) obtained from the high-cycle fatigue tests of the Ti-xMo-2Fe (x = 3.4, 5, 9.2 wt.%) alloys under various stress conditions are shown in Figure 12. At the maximum stress of 550 MPa during the test, 32 SW fractured at 1.9 $\times 10^{6}$ rotations, but at the minimum stress of 450 MPa, 32 SW did not fracture even at the fatigue limit of more than $10^{7}$. At the maximum stress of 500 MPa, 52 SW fractured at 6.2 $\times 10^{6}$ rotations, but at the minimum stress of 476 MPa, 52 SW did not fracture even at the fatigue limit of more than $10^{7}$. By contrast, at the maximum stress of 450 MPa during the test, 92 SW fractured at 9.7 $\times 10^{4}$ rotations, and at the minimum stress of 350 MPa, 92 SW fractured at 1.3 $\times 10^{6}$ rotations. The results of the high-cycle fatigue tests showed that the 52 SW and 92 SW alloys had the highest and lowest fatigue strengths, respectively. A previous study reported that fatigue strength is related to the thickness of the α-lath, where the thinner the α-lath, the greater is the fatigue strength [39]. Therefore, as the α-lath width was 1.35, 0.93, and 0.48 µm in the 32 SW, 52 SW, and 92 SW designed alloys, respectively, 52 SW was thought to have higher fatigue strength than 32 SW because of its thinner α-lath, thereby suppressing the formation and progression of microcracks. However, 92 SW had the lowest fatigue strength even though it had a thinner α-lath than that of 52 SW. The ω phases exist as hard particles in a matrix, and secondary slip is locally activated near the ω phase. Therefore, the ω phase quickly reaches the critical stress for forming voids even under a small amount of deformation and thus exhibits low resistance to necking and damage [38,40]. Accordingly, 92 SW fractured because of the relatively low fatigue strength caused by the ω phase.

**Figure 13:**
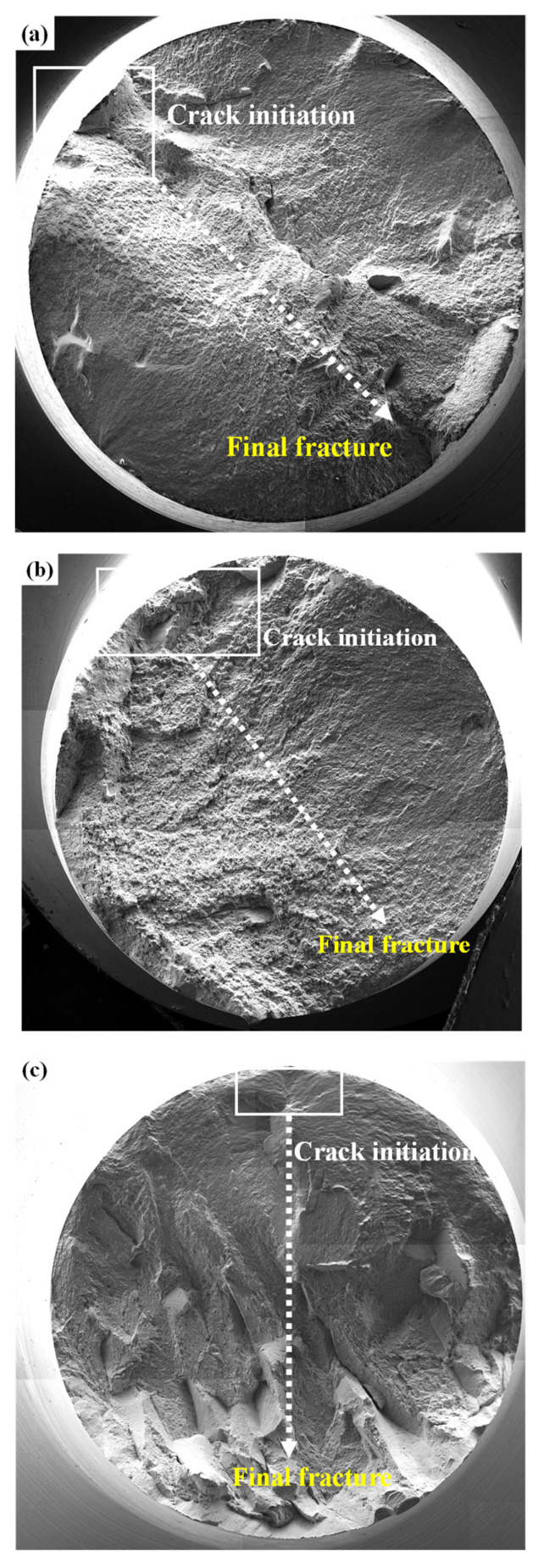
SEM fractographs of the as-swaged Ti-xMo-2Fe alloys after high-cycle fatigue test; (**a**) 32 SW, (**b**) 52 SW, and (**c**) 92 SW.

Figure 13 shows the fracture surfaces of the fractured high-cycle fatigue specimens. In the case of the 32 SW and 52 SW specimens, which showed high fatigue strengths of 450 MPa or greater, the cracks began at the top left and spread to the bottom right until final fracture occurred. In addition, dimples, which are characteristic of ductile fracture, were found throughout the specimens. Unlike the other specimens, cleavage fracture was observed throughout 92 SW, which is a characteristic of brittle fracture and confirms the previously described phenomenon of fracture prior to the yield point.

Consequently, the superior tensile and high-cycle fatigue properties of the 52 SW alloy can be understood in terms of microstructural effects associated with the formation of stress-induced martensite (α″). The β → α″ transformation provides an additional deformation mechanism similar to transformation-induced plasticity (TRIP) during loading, which helps to delocalize local strain, increase uniform deformation, and thereby improve elongation. In addition, the formation of α″ laths refines the effective grain/lath size, which enhances the strength according to the Hall–Petch relationship while simultaneously alleviating excessive strain concentration at specific grain boundaries or interfaces. These effects operate in a similar manner under high-cycle fatigue loading, where deformation is more widely distributed throughout the specimen and stress concentration at particular defects or interfaces is suppressed, thereby delaying crack initiation. In contrast, the ω phase observed in the 92 SW alloy is a nanoscale, very hard and brittle precipitate that strongly hinders dislocation motion and provides local sources of stress concentration, thereby causing premature crack initiation during tensile deformation and promoting both crack initiation and propagation under high-cycle fatigue loading. Therefore, the opposite roles of SIM-induced α″ formation in the 52 SW alloy and ω phase precipitation in the 92 SW alloy can be considered responsible for the pronounced differences in tensile and fatigue properties between the two alloys.

## 4. Conclusions

This study designed Ti-Mo-Fe metastable β-Ti alloys under various amounts of Mo and examined changes in the alloy microstructures and mechanical properties caused by SIM transformation resulting from the cold swaging process. The main results of this study can be summarized as follows.

1. The 32 SW alloy consisted of the α and β phases. First, the $\alpha^{\prime \prime }$ phase for the 52 SW alloy formed as a result of SIM transformation. Furthermore, 92 SW, which had the highest Mo content, contained the $\alpha^{\prime \prime }$ and ω phases. This was because β stability increased with Mo content and thus altered the phase transformation mechanism.
2. 52 SW exhibited the highest tensile strength (1179.4 MPa) and elongation (3.3%) due to the formation of the $\alpha^{\prime \prime }$ phase with increased Mo content. The $\alpha^{\prime \prime }$ phase also occurred in 92 SW due to SIM transformation. However, 92 SW experienced brittle fracture prior to the yield point as a result of the ω phase.
3. The greater the amount of Mo, the thinner the α-lath, and this affected fatigue strength. Analytical results of the high-cycle fatigue properties showed that 52 SW had the highest fatigue limit. In contrast, 92 SW, which had the narrowest α-lath interval, was expected to have a high fatigue limit. In fact, it had the lowest fatigue limit (more than $10^{6}$ cycles at less than 350 MPa) due to the formation of the ω phase.
4. Using MoE, e/a, and the d-electron alloy design method, we were able to predict the designed alloy transformation mechanisms and constituent phases, where the 52 SW alloy showed the highest tensile strength and highest fatigue limit (476 MPa). Finally, because the high-cycle fatigue results were obtained from a limited number of specimens and the difference in fatigue life between 32 SW and 52 SW was small, further fatigue testing with an increased number of specimens is required to statistically confirm the observed trend.

## Figures and Tables

**Table 1:** The comparison of several Ti-xMo-2Fe alloys, MoE, $\beta_{tr}$, $\bar{Bo}$, $\bar{Md}$ and e/a.

	MoE	$\beta_{tr}$	$\bar{Bo}$	$\bar{Md}$	e/a
Ti-3.4Mo-2Fe	9.2	818.9	2.412	2.792	4.105
Ti-5Mo-2Fe	10.8	803.7	2.408	2.794	4.122
Ti-9.2Mo-2Fe	15	763.8	2.397	2.801	4.178

**Table 2:** Volume fractions of various phases.

	$V_{f,\alpha }$	$V_{f,\alpha^{\prime \prime }}$	$V_{f,\beta }$	$V_{f,\omega }$
32 SW	66.4	-	33.6	-
52 SW	23.9	57.2	18.9	-
92 SW	12.6	41.5	43.9	2

**Table 3:** Tensile properties of as-swaged Ti-xMo-2Fe alloys.

	Yield Strength (MPa)	Ultimate Strength (MPa)	Elongation (%)
32 SW	969.0	993.2	1.7
52 SW	1146.4	1179.4	3.3
92 SW	-	565.8	1.1

## Data Availability

The original contributions presented in this study are included in the article. Further inquiries can be directed to the corresponding author.
